# Horizon scan of rapidly advancing coral restoration approaches for 21st century reef management

**DOI:** 10.1042/ETLS20210240

**Published:** 2022-02-04

**Authors:** David J. Suggett, Madeleine J.H. van Oppen

**Affiliations:** 1 University of Technology Sydney, Climate Change Cluster, Faculty of Science, Ultimo, NSW, Australia; 2 School of Biosciences, The University of Melbourne, Melbourne, VIC, Australia; 3 Australian Institute of Marine Science, Townsville, QLD, Australia

**Keywords:** assisted evolution, coral, cryopreservation, management, propagation, restoration

## Abstract

Coral reef restoration activity is accelerating worldwide in efforts to offset the rate of reef health declines. Many advances have already been made in restoration practices centred on coral biology (coral restoration), and particularly those that look to employ the high adaptive state and capacity of corals in order to ensure that efforts rebuilding coral biomass also equip reefs with enhanced resilience to future stress. We horizon scan the state-of-play for the many coral restoration innovations already underway across the complex life cycle for corals that spans both asexual and sexual reproduction — assisted evolution (manipulations targeted to the coral host and host-associated microbes), biobanking, as well as scalable coral propagation and planting — and how these innovations are in different stages of maturity to support new 21st century reef management frameworks. Realising the potential for coral restoration tools as management aids undoubtedly rests on validating different approaches as their application continues to scale. Whilst the ecosystem service responses to increased scaling still largely remain to be seen, coral restoration has already delivered immense new understanding of coral and coral-associated microbial biology that has long lagged behind advances in other reef sciences.

## Introduction

Coral reef management is at a historical turning point. Management strategies until now have centred on conservation through marine protected areas alongside mitigation of local and regional stressors, such as declining water quality from coastal urbanisation, and intensive agriculture and industry in reef catchments. Whilst these strategies undoubtedly slow the decline of reef health [1], reef degradation continues to accelerate under climate change [2]. Even under a scenario where global emissions can be reduced immediately, reefs will likely continue to be exposed to frequent climate stress events for the coming decades (e.g. [3,4]), leaving reef dependent communities — almost a billion people — with an entirely uncertain future and in the hands of global climate policy makers [5]. The need for additional management options that can be implemented now has created a global drive for practical local action whilst critically tackling climate change [5–7].

The last decade has witnessed acceleration of reef restoration activities around the world [8–10], often through local community-driven programs looking to boost rates of coral recovery. For example, recent activities range from re-planting of naturally at-risk coral material — such as broken fragments or detached colonies (i.e. fragments- or colonies- of opportunity) — to propagating colonies of critical species of interest using *in situ* or land-based nurseries. To develop more standardised practice as well as catalyse innovative tools, activities have become increasingly networked via regional or global bodies (e.g. the Coral Restoration Consortium) with goals to more systematically and collectively build knowledge [11], whilst also adopting core principles from more well established terrestrial and coastal restoration fields [12–14] (Box 1). In doing so, building a more robust scientific process has galvanised this movement to transform the scale, feasibility and hence cost-effectiveness needed to solidify reef restoration as tangible management action [7,15]. Of particular focus has been the rapid rise of practices looking to augment the high adaptive state and capacity of corals (*sensu* [16–19]) and integrating this into rapidly advancing propagation and planting (or deployment) methods, so that efforts to rebuild coral biomass also equip reefs with enhanced resilience to future stress.

Box 1. **General principles for viability and development of coral reef restoration: ten ‘key rules' for reef restoration programs ([14]; see also [11,12,105])**

1. **Protect existing reefs first:** Minimise impacts that increases the extent of restoration required.
2. **Work together:** Collective action across practitioners as well as across disciplines is required to build scalable solutions.
3. **Maximise biodiversity to reach recovery goals:** Biodiversity — including genetic diversity of individual species — is a key factor regulating reef resilience to stress.
4. **Select appropriate reef areas:** Not all areas can (or should) be restored, and requires consideration of ecological, economic and cultural values.
5. **Use natural regeneration where possible:** Adopt-nature-based solutions — as well as identifying barriers slowing natural recovery — is essential where possible.
6. **Select species to maximise biodiversity:** Adopt a species priority framework relative to ecosystem service recovery goals.
7. **Use resilient material:** Rebuilding ‘reefs of tomorrow' with coral that is more tolerant to anthropogenic stressors (climate change, lower water quality etc.).
8. **Plan ahead for infrastructure, capacity and (coral) supply:** Ensuring access to sufficient and appropriate material, and longevity of monitoring out-planting needed to gauge ecological success over time.
9. **Learn by doing:** Outcomes are often context specific and so practices require ‘fast-fail' exercises to tailor, as well as fully reporting ‘failures’.
10. **Make it pay:** Ensure financing is in place to deliver goals — in particular where coral is not a CO_2_ sink and therefore does not directly fall under blue carbon market economies.

**Figure 1.**
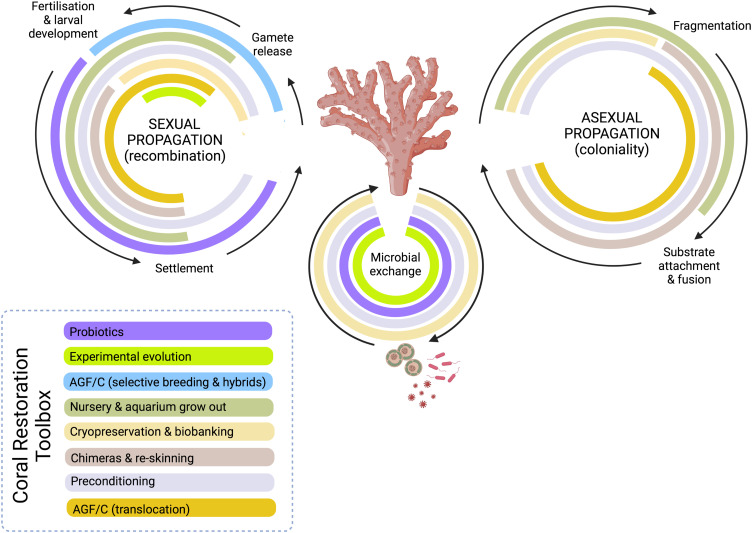
Mapping innovations in coral restoration to the coral life cycle. Innovations are categorised (as per the main text) as assisted evolution methods: Targeting coral host (Manipulations targeted at the coral host) through Assisted Gene Flow/Colonisation (AGF/C) using either selective breeding (intra- and intra-hybridisation) via sexual reproduction or translocation of adult (or larval) material; chimeras and re-skinning of adult material; or preconditioning. Targeting host-associated microbes using either experimental evolution or probiotics. Also, biobanking of live adult material or cryopreservation of coral gametes or associated microbes (Cryopreservation and biobanks), as well as *in situ* nursery- and aquarium-based propagation and grow-out (Propagation and planting). Created with BioRender.com.

Several disciplines are now captured within the new field of coral reef restoration, including geoengineering (e.g. cloud brightening [20]; rubble stabilisation [21]) and applied ecology (e.g. algal removal [22]; connectivity modelling [23]), but here we specifically focus our horizon scan on restoration practices centred on coral biology, and hence coral restoration. We review the state-of-play for the many coral restoration innovations already underway across the complex life cycle for corals (Figure 1) — which encompasses both asexual and sexual reproduction — and how these approaches increasingly show promise as routine reef management actions for the 21st century.

### Assisted evolution

Most coral restoration efforts have historically used coral genotypes from the sites in need of restoration or from nearby — less affected and healthier — sites. However, adaptation to the local prevailing environmental conditions means that such genotypes inevitably carry low survivorship to further disturbance events of similar nature to those that caused severe degradation initially. This is of particular concern for climate change-driven summer heatwaves, which are predicted to become more frequent, protracted and severe over the coming decades [3,4], causing coral bleaching and accelerating disease outbreaks [24]. Use of coral stock with greater tolerance is therefore required for better restoration outcomes. Bioengineering approaches such as selective breeding and microbiome manipulation have been used across biological systems to improve desirable traits, including yield and nutritional value of fruits and seeds in crops, growth rate and meat quality in livestock and aquaculture species, and pathogen and environmental stress resistance in crops and plantation trees. More recently, such approaches have been used for the conservation of genetic diversity and species [25], prevent disease in wildlife [26], and for ecosystem restoration [27]. In corals, bioengineering methods have only recently begun to be explored, and are referred to as **assisted evolution** (AE) [16]. AE is an umbrella term for a suite of manipulations aimed either at the coral host animal or its associated microbiota and defined as the acceleration of naturally occurring evolutionary processes to enhance certain traits, where current focus of coral AE is mostly on augmenting resilience to climate stress [17,19].

### Manipulations targeted at the coral host

**Assisted gene flow** and **assisted colonisation** (or assisted migration) refer to the translocation of individuals within and beyond their distribution range, respectively. The rationale is that local adaptation has resulted in different alleles and/or allele frequencies at loci underpinning thermal tolerance, and that adaptation to rising temperatures can be enhanced by translocating individuals harbouring these adaptive alleles. Following translocation, it is anticipated that transplants interbreed with the native population and that heat tolerance alleles will be incorporated into the native genetic background. Translocation can be achieved with both early life stages or adults, within a reef [28], to a nearby reef [29–31] or over larger distances within [32] or between regions [33]. Findings so far suggest transplants largely retain the tolerance ranking exhibited at their native reef site.

Rather than translocating comparatively thermally tolerant individuals, such individuals may instead be cross-bred *ex situ* with individuals of lower tolerance inhabiting cooler reefs, followed by deployment of intraspecific hybrid offspring onto depauperate (cooler) reefs for restoration in anticipation of future heatwaves [34–36]. Additionally, considerable variation in thermal tolerance has been uncovered among colonies on the same reef [29,36], enabling **selective breeding** for production of more thermally tolerant offspring for restoration [37]. While the genetic contribution to heat tolerance (i.e. heritability) varies with specific life stages and across thermal tolerance traits, heritability appears high for most traits [38] and thermal tolerance of offspring tends to be enhanced if at least one of the parents is comparatively thermally tolerant [35,36,39,40]. Cross-breeding between — rather than within — species increases genetic diversity and creates new allele combinations in **interspecific hybrid** offspring (Figure 1), which can promote environmental stress tolerance [41–43]. Indeed, first generation interspecific hybrids have been shown to grow and survive as well as or better than purebreds of their parental species under both ambient and future predicted reef conditions [44]. In all cases, it is critical that genetic diversity is maintained to ensure populations remain resilient to diverse stress factors.

**Figure 2.**
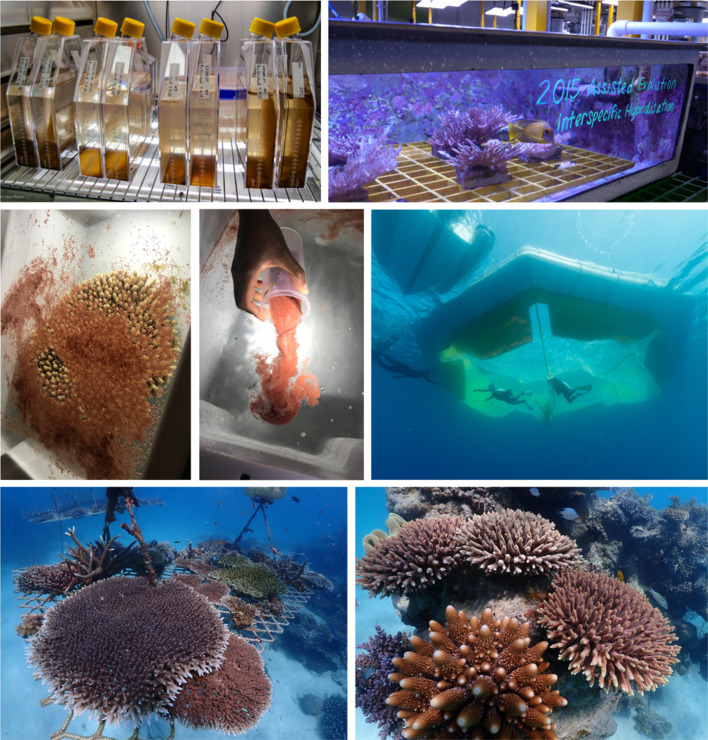
Examples of coral restoration research and practice. (Top Left) Many algal symbionts of corals can be isolated and brought into pure culture for various AE activities; for example, enhancement of the upper thermal tolerance limits of the cultured strains by subjecting them to increasing culture temperatures (© Australian Institute of Marine Science (AIMS)). (Top right) Different *Acropora* species have been crossed to produce interspecific hybrids with increased climate resilience relative to their parental species (© AIMS). (Middle left, middle) Collecting coral spawn (*Acropora* sp.) and cleaning egg-sperm bundles for controlled fertilisation, approaches needed for both AE activities and larval enhancement-based propagation. (Middle right) Establishing and inspecting in-water larval rearing ponds for propagating spawn slicks on the reef (© Juergen Freund). (Bottom left) Colonies of *Acropora* sp. propagated from fragments after 12–18 months using in water nurseries (© John Edmondson/Wavelength). (Bottom right) Colonies of *Acropora* sp. established 12–18 months from replanting fragments of opportunity back to the reef (© John Edmondson/Wavelength).

A relatively underexplored manipulation of coral host genetics is through active formation of **chimeras**, entities comprised of cells from two or more individuals. Fusion of larvae at settlement, or when growing juvenile colonies physically contact one another, can result in an immediate size increase and associated increased survivorship of the young coral [45,46]. Chimeras consist of two or more genotypes and so have enhanced genetic diversity compared with non-chimeric colonies, as well as elevated physiological diversity, and potentially increased tolerance to stressors such as elevated temperature [46,47]. However, stable tissue fusion usually only occurs between closely related individuals (e.g. [48]); thus, to achieve high numbers of chimeras, rearing and settling of individual larval families prior to field deployment would be required, which may prove challenging and costly at scales relevant to the amount of biomass needed for restoration (Propagation and planting).

**Preconditioning** is an AE technique whereby an environmental stress memory is induced by exposing corals to sublethal stress to drive enhanced stress tolerance [49]. Stress memory in corals was first discovered in east-facing surfaces of small massive corals that bleached during a summer heatwave whereas west-facing surfaces — receiving higher light doses — did not [50]. Whilst some studies have since demonstrated that moderate thermal [51–53] and acidification [54] stress can also enhance tolerance to subsequent thermal stress, others have shown pre-conditioning is not a guarantee to boosting stress tolerance [55]. On balance, given the potential for enhancing stress tolerance through careful environmental exposure, preconditioning seems useful for reef restoration during the larval and early recruit rearing phase of sexually produced coral stock, but also for asexual adult propagation by carefully choosing the location of *in situ* nurseries (Propagation and planting).

### Manipulations of coral-associated microbes

Host-associated microbes play critical roles in holobiont physiology, stress tolerance and adaptation to environmental change [56]. Corals associate with many groups of microbes [19], but the current targets for AE are the microalgal symbionts housed within coral gastrodermal cells (dinoflagellates in the family Symbiodiniaceae), and bacteria that reside in most coral compartments. Symbiodiniaceae is a species-rich family comprising thermally tolerant and sensitive species, and coral holobiont thermal tolerance is largely contingent on the Symbiodiniaceae community it harbours (e.g. [57]). Many Symbiodiniaceae species have narrow host ranges (e.g. [58]); consequently, pairing coral with naturally more heat-tolerant Symbiodiniaceae species — but that are heterologous — does not always result in compatibility. A possible solution is to augment thermal tolerance of homologous Symbiodiniaceae isolates *ex hospite* (in culture) via **experimental evolution** [59] (Figure 1), followed by the reintroduction of the heat-evolved isolates into coral. This procedure has already been shown to increase thermal tolerance in coral larvae [60] and juveniles [61]. Thus, for reef restoration, aposymbiotic coral early life stages could be inoculated with heat-evolved symbionts to boost their thermal tolerance prior to deployment onto degraded reefs. Experimental evolution is highly amenable to coral-associated bacteria suggesting it also has value to increase coral climate resilience and therefore also useful for coral restoration [62].

Bacterial **probiotics** have been widely and successfully used to treat disease and enhance traits including environmental stress tolerance in biological systems other than corals [63,64]. How individual bacterial species govern coral holobiont functioning is not well understood, but growing evidence suggests key roles in nutrient cycling, defence against microbial pathogens and possibly thermal tolerance (e.g. [65]), prompting interest in developing bacterial probiotics to offset the negative impact of transient heat stress on coral health [66]. Inoculation of corals with cultured bacteria or whole microbiomes isolated from healthy coral can modify the coral-associated bacterial communities, although changes may not be temporally stable [67–70]. In recent experiments, inoculation with a bacterial cocktail consisting of a small number of strains [68,70] or a microbiome transplant [69] lowered heat stress susceptibility relative to no-inoculum controls. Whilst such findings are promising, these studies are yet to account for the fundamental problem that the inoculum merely serves as food — and hence greater metabolic resources to drive stress resistance [71] — as opposed to actual bacterial traits being responsible for the enhanced tolerance.

### Cryopreservation and biobanks

By analogy to seed banks for terrestrial restoration, **coral and coral-associated microbial biobanks** are necessary to safeguard diversity against uncertain future environmental conditions. Developing and scaling infrastructure to ensure a living repository is therefore a core requirement for coral restoration (e.g. [14]; Box 1). Whilst land-based aquarium systems — in particular those that already support propagation practices (Propagation and planting) — carry potential to support live material biobanking (e.g. ‘World Coral Conservatory', [72]), **cryopreservation** is most desirable to reduce maintenance and transportation footprints to conserve extensive genetic diversity. Huge advances have already been made in effective cryopreservation of sperm and embryos for >30 coral species from both the Caribbean and Indo-Pacific [73], and in demonstrating the utility of cryopreserved coral sperm for intraspecific hybridisation [74]. Major efforts are also underway to establish cryo-banks of coral-associated bacteria [75], given their roles in AE (Manipulations of coral-associated microbes). Attempts to cryopreserve Symbiodiniaceae have been confounded by long-standing freeze-thaw challenges for dinoflagellates, but even so has been shown successful for a number of isolates spanning multiple genera [76] and phenotypes (e.g. thermally tolerant, [77]). Bio-bank proposals have built on these cryopreservation successes [78] and will no doubt prove powerful in aiding coral restoration activities in future where natural coral abundance and diversity is likely to be diminished.

### Propagation and planting

Capacity to effectively propagate and (re)plant coral is a (arguably, *the*) fundamental operational bottleneck to ultimately rebuild biomass back to reefs. Adult coral is commonly propagated asexually using ***in situ* nurseries** as well as **land-based aquaria** [8] (Figures 1 and 2). *In situ* nursery practices have advanced by tailoring species choice, location to optimise growth, and/or design to improve cost-effectiveness [79–81], enabling both long term housing of parental stocks as well as short term ‘grow out' of material (e.g. [82]). New tools that can rapidly discriminate genotypes (or phenotypes) are particularly essential to maximise diversity in the propagation of any given species [83–85]. Nurseries may fast-track growth to reach sexual maturity sooner and therefore also serve to kick-start new reproductively competent stock back to the reef [86]. Land-based aquaria provide many benefits, ranging from controlled coral growth environments to facilitating broader public engagement [87] but will need to advance in technologies for clean energy and mobility to ensure sustainable and scalable operations. Upsizing capacity inevitably carries risks commonly encountered in large scale mono-culturing, notably disease [88]. As such, treatments with probiotics (Manipulations of coral-associated microbes) for disease prevention and to maximise growth and survival in captivity — that have become increasingly common-place in aquaculture — may become a key feature of propagated coral in future. Employing ‘supplements’ beyond probiotics — notably **nutraceuticals (and prebiotics)** — remains largely unexplored within coral restoration despite decades of research identifying how specific resources enhance coral tolerance to suboptimal environmental conditions (e.g. [71]).

Whilst asexual propagation can be conducted year-round, restoration methods also rely on sexual propagation to yield large numbers of offspring, ensure genetic diversity, and use of some AE interventions ([17]; Assisted evolution and Manipulations targeted at the coral host). This need for sexually produced coral stock has led to a resurgence of research to understand coral reproductive life cycles [89]. Four major modes of sexual reproduction have been identified in coral — but the majority of species are broadcast spawners that reproduce *en mass* once or twice per year [89]. Many challenges still remain to rear offspring from brooders [89,90]. Growing records of coral reproduction timing in nature [91] now enable practitioners to better target spawning events, whilst improved knowledge of reproduction cues has led to innovative land-based systems to induce multiple spawning events *ex situ* [92], allowing limitations of natural timings to be overcome. A critical focus of larval-based methods is to maximise larval production, survival and resilience. However, the biggest challenge with sexual-based propagation remains improving the inherently low survivorship of larvae or early recruits returned to the reef. Deployment methods range from **larval settlement devices** that support early growth of corals (e.g. [93]) on the reef, to **mass larval seeding** (e.g. [90,94]). In all cases, survival and growth from larva to adult coral remains low, and unlocking the biology of coral early life stages at the reef scale where it exists, i.e. micro-scale physico-chemical gradients as well as cryptic ecological interactions, remains a critical goal towards more effective sexual-based propagation.

**Planting** (‘out-planting' or ‘deployment’) adult coral material (fragments, colonies) or newly settled larvae is a resource- and time-intensive restoration step. Heterogeneous topographies and habitats of reef systems make random deployment highly ineffective. Fixing material to the reef has increasingly evolved towards rapid and high-throughput chemical [95] or physical attachment, e.g. clips or plugs [96] (Figure 1); and in the case of chemical-based approaches, towards new bio-adhesives (e.g. [97]) to overcome limitations of artificial glues. In all cases, research has turned to addressing a key aspect of coral biology that has long remained obscure - how material actually attaches to substrates - to further optimise attachment protocols (e.g. the first ‘coral attachment model'; [98]). Practicalities of attachment are equally critical in innovations such as micro-fragmentation to establish rapid growth of outplants (and notably of massive corals; [99]) needed for ‘re-skinning' of large substrate areas.

Together, these various steps have already increased the scale — and hence cost-effectiveness — of planting over the past few years [8,96,100,101], representing an important feedback-loop: Evaluating the outcome of planting at increasing scale is a critical step needed to optimise future practice aimed at enhancing survivorship and the scale of restoration initiatives [102]. Such incremental gains — through relatively small and localised, but collective, activity — are thus the essential pathway to building big restoration outcomes [103].

## Outlook for coral restoration within 21st century management

Addressing the root causes of human disturbances such as climate warming and deteriorating water quality, and targeted conservation measures will be essential for healthy coral reefs to persist into the next century; however, it is inevitable that coral restoration-centred activities become an increasingly dominant aid to regional reef management portfolios in the short- to mid-term, particularly where local stakeholder motivation intensifies to actively steward improved reef health [5,7]. Consequently, the feasibility of coral restoration approaches rests on advancing in parallel research and development (across the ‘risk-reward continuum', [104]; Manipulations targeted at the coral host, Manipulations of coral associated microbes, Cryopreservation and biobanks) and cost-effective implementation ‘on the ground’ [100,101] (Propagation and planting; Figure 1 and 2). This in turn is critical to position reef managers with the operational capacity to act, but also the means to inform what combination of approaches best suit different reef sites of alternate conditions and/or service value [105].

Within a 21st century reef management framework, acceleration of coral reef restoration practices will undoubtedly be fuelled via the UN Decade of Ecosystem Restoration [15,106], which launched in June 2021. However, translating this acceleration of practices into tangible and valid management aids still rests on ensuring three key inter-dependent factors (adapted from [11,103]):

**(1)** **Advancing the scale of practice to achieve ecological outcomes.** Whilst scalable and cost-effective coral propagation and planting activity is increasingly evident (e.g. [81,96,102,107]), the extent with which this yields ecological and social gains remains uncertain [105]. That said, some efforts have now been established long enough to see how restoration of coral cover can enhance associated fish communities in the Indo-Pacific [100,101,107,108]. Similarly, restored coral populations in the Caribbean and Indo-Pacific have recently been observed spawning for the first time [109], and hence completing the cycle of asexually propagated material to sexually reproducing populations (Figure 1). Ensuring these scales of implementation continue to return over time rests on increasing resilience of planted material against future stressors (Manipulations targeted at the coral host, Manipulations of coral associated microbes, Cryopreservation and biobanks).
**(2)** **Building social support to motivate political and industry buy in.** Reef livelihoods and cultures rely on healthy sites, and so industries such as tourism — but also traditional owners — already see the value of coral restoration to enhance existing stewardship activities (e.g. Great Barrier Reef [10,79,96,110]). However, building social licence is not a given, or indeed straightforward. Scepticism of reef restoration practices are understandably firmly in place, where investment returns seem low and are viewed as a political smokescreen against taking bigger action on climate change. Whilst such scepticism often under-recognises the immense social value of pro-active stewardship as well as the knowledge gains made through novel research, it is a powerful motivator to ensure restoration efforts are validated, held accountable and continue to innovate.
**(3)** **Overcoming economic inertia to value the environment.** Mitigation strategies almost certainly rest on prioritising economic capital into a low carbon economy and safeguarding reef systems. However, investing into more sustainable use of reef systems is required as the human footprint continues to grow. Diverse funding streams — spanning philanthropy, corporate investment and Government grants — have provided the capital to initiate coral restoration programs (e.g. [104]). However, transitioning restoration efforts towards sustained financing initiatives (e.g. [111,112]) — that importantly support coupled research and operational activities needed to advance successes (e.g. [110]) — is critical to ensure longevity to realise what are often long-term proposed ecological and social goals.

Ultimately cost is no doubt a fundamental constraint for local uptake of many activities outlined here and a major barrier to equitable access and/or deployment at relevant scales. Investment therefore needs to prioritise mobilisation of restoration tools for adoption by the global stakeholders (locations) most in need. In turn, local governance will need to be pre-armed with capacity to rapidly activate adoption within risk-assessed 21st century management frameworks to capitalise on the rapidly advancing financing opportunities for coral and reef restoration.

## Conclusion

Coral restoration is a rapidly emerging discipline within reef sciences, and approaches that exploit the natural adaptive capacity of corals across sexual and asexual life cycle phases are already in various stages of maturity towards more diverse reef management frameworks. Feasibility and scalability by which approaches are applied are advancing continuously. Whilst significant steps and time-frames are still required to understand how increasing activities in reef systems are impacting key ecosystem service values, global coral restoration efforts have already catalysed new knowledge of coral and coral-associated microbial biology that has progressed more slowly compared with advances in other reef sciences (e.g. ecology).

## Summary

- Restoration practices centred on coral biology (coral restoration) have rapidly accelerated via a global drive for practical local action whilst critically tackling climate change.
- Many methods are in various stages of maturity, all of which encompass the high adaptive capacity of corals across their complex life cycle where proliferation occurs through sexual reproduction and asexual (colonial) propagation.
- Innovations in assisted evolution-based tools, biobanking and propagation practices are transforming understanding of coral biology that has long lagged behind advances in other reef sciences (e.g. ecology).
- Efforts continue to deliver increases to the scale of feasible coral restoration *in situ*, but how these deliver ecosystem service outcomes remains to be seen, requiring longevity in economic inertia and social gains, and ensuring restored coral carries increase resistance to on-going environmental stress.

## Abbreviations

AE, assisted evolution; AIMS, Australian Institute of Marine Science.
